# BRAF^V600E^ inhibition stimulates AMP-activated protein kinase-mediated autophagy in colorectal cancer cells

**DOI:** 10.1038/srep18949

**Published:** 2016-01-11

**Authors:** Toshinori Sueda, Daisuke Sakai, Koichi Kawamoto, Masamitsu Konno, Naohiro Nishida, Jun Koseki, Hugh Colvin, Hidekazu Takahashi, Naotsugu Haraguchi, Junichi Nishimura, Taishi Hata, Ichiro Takemasa, Tsunekazu Mizushima, Hirofumi Yamamoto, Taroh Satoh, Yuichiro Doki, Masaki Mori, Hideshi Ishii

**Affiliations:** 1 Department of Frontier Science for Cancer and Chemotherapy, Osaka University, Graduate School of Medicine, 2-2, Yamadaoka, Suita, Osaka, 565-0871, Japan; 2 Department of Gastrointestinal Surgery, Osaka University, Graduate School of Medicine, 2-2, Yamadaoka, Suita, Osaka, 565-0871, Japan; 3 Department of Cancer Profiling Discovery, Osaka University, Graduate School of Medicine, 2-2, Yamadaoka, Suita, Osaka, 565-0871, Japan; 4 Department of Molecular Pathology, Osaka University Graduate School of Medicine and Health Science, 1-7, Yamadaoka, Suita, Osaka, 565-0871, Japan

## Abstract

Although BRAF^V600E^ mutation is associated with adverse clinical outcomes in patients with colorectal cancer (CRC), response and resistance mechanisms for therapeutic BRAF^V600E^ inhibitors remains poorly understood. In the present study, we demonstrate that selective BRAF^V600E^ inhibition activates AMP-activated protein kinase (AMPK), which induces autophagy as a mechanism of therapeutic resistance in human cancers. The present data show AMPK-dependent cytoprotective roles of autophagy under conditions of therapeutic BRAF^V600E^ inhibition, and AMPK was negatively correlated with BRAF^V600E^-dependent activation of MEK-ERK-RSK signaling and positively correlated with unc-51-like kinase 1 (ULK1), a key initiator of autophagy. Furthermore, selective BRAF^V600E^ inhibition and concomitant suppression of autophagy led to the induction of apoptosis. Taken together, present experiments indicate that AMPK plays a role in the survival of BRAF^V600E^ CRC cells by selective inhibition and suggest that the control of autophagy contributes to overcome the chemoresistance of BRAF^V600E^ CRC cells.

Although outcomes in patients with colorectal cancers (CRC) have improved over the last decade, poor prognoses remain for some subtypes of CRC^1^. In particular, mutations in valine 600 (V600) of the BRAF oncogene occur in approximately 7% of all human cancers, including approximately 10% of CRC^1^,^2^. Moreover, BRAF mutations are associated with adverse clinical outcomes in patients with CRC, with a 70% increase in mortality in patients with metastatic CRC harboring BRAF^V600E^ mutations compared with those carrying wild-type BRAF^3^,^4^. Therefore, novel therapeutic strategies for patients with BRAF mutant CRC are critically needed.

Although a selective RAF inhibitor was recently approved by the Food and Drug Administration for the treatment of metastatic melanomas harboring BRAF^V600E^ mutations, response rates to selective BRAF inhibitors vary between tumor types. While selective BRAF inhibitors have produced response rates of approximately 50%–80% in patients with BRAF^V600E^ mutant melanomas^5^, a selective BRAF inhibitor alone has proven disappointingly ineffective in CRCs harboring BRAF^V600E^ mutations. Multiple studies have investigated the underlying mechanisms of resistance of BRAF^V600E^ CRC to selective BRAF inhibitors, including KRAS and BRAF amplifications and MEK1 mutations^6^. Other studies have shown that EGFR-mediated reactivation of the mitogen-activated protein kinase (MAPK) pathway, PIK3CA mutations, and PTEN loss may also contribute to selective resistance to BRAF inhibitors^7^. However, the relative correlations with these resistance mechanisms and clinical outcomes remain poorly understood. Therefore, elucidating the underlying mechanisms of resistance to selective BRAF inhibitors may lead to new therapeutic strategies for CRCs harboring the BRAF^V600E^ mutation.

Autophagy has been described as a mechanism of resistance for cancer cells under conditions of therapeutic stress in numerous human cancers, including CRC. Autophagy is an intracellular bulk degradation system in which cytoplasmic components, including organelles, are directed to the lysosome/vacuole by a membrane-mediated process^8^. Autophagy is thought to be initiated under nutrient-limited conditions by a conserved kinase complex containing the unc-51-like kinase 1 (ULK1) and ULK2 and the subunits autophagy-related gene 13 (Atg13) and FAK family kinase-interacting protein of 200 (FIP200)^9^. Although autophagy is activated under chemotherapy or radiation stresses^10^,^11^, subsequent influences on cancer cell death or survival remain controversial. However, numerous reports indicate that the activation of autophagy promotes cancer cell survival after exposure to chemotherapy or radiation therapy and inhibition of autophagy can be a valuable strategy for cancer therapy.

Autophagy is a complicated regulatory process that involves numerous upstream regulating signaling pathways, including the PI3K-Akt-mammalian target of rapamycin (mTOR) pathway; liver kinase B1 (LKB1)-AMP-activated protein kinase (AMPK)-mTOR pathway; and p53, Beclin1, and Bcl-2 pathways^12^ and, to a limited extent, MAPK signaling pathway. Whether autophagy is required for BRAF^V600E^ CRC remains unclear, evidence suggests that it is important for BRAF^V600E^ melanomas^13^,^14^. Interestingly, previous studies report a molecular relationship between LKB1-AMPK and RAF-MEK-ERK pathways in melanomas harboring the BRAF^V600E^ mutation^15^,^16^. However, to the best of our knowledge, no previous studies have examined the molecular linkage between the BRAF^V600E^ mutation and selective BRAF inhibitor-induced autophagy in BRAF^V600E^ CRC. Considering the potential roles of AMPK-related cellular signaling pathways, such as the MEK-ERK pathway, we hypothesized that AMPK interacts with the MEK-ERK pathway to induce autophagy in BRAF^V600E^ CRC.

In the present study, we report elevated levels of autophagy after exposure to selective BRAF inhibitors in BRAF^V600E^ CRC cells. Subsequently, the roles of selective BRAF inhibitor-induced autophagy, the effects of autophagy inhibition by small-interfering RNAs (siRNAs) or a pharmacological inhibitor, and the mechanistic link between BRAF^V600E^ mutation and autophagy in BRAF^V600E^ CRC cell lines were studied. Our findings indicate that selective BRAF inhibitor-induced AMPK phosphorylation coordinates control of autophagy and tumor chemoresistance in BRAF^V600E^ CRC cells.

## Experimental Procedures

### Reagents and antibodies

Selective BRAF inhibitors PLX4032 (also known as Vemurafenib, AXON Medchem, catalogue #1624; AdooQ BioScience Catagog Num A10739) and PLX4720 (AXON Medchem, #1474) and Chloroquine (CQ) (Focus Biomolecules, #10-2473; SIGMA-ALDRICH, C6628) were used. The antibodies for Western blotting are as follows: the microtubule-associated protein 1 light chain 3 (LC3) (Cell Signaling Technology, CST, #2775); anti-Atg13 (CST, #13468); anti-Atg7 (CST, #2631); anti-phospho-mTOR (Ser2448) (CST, #2971); anti-mTOR (CST, #2972); anti-phospho-AMPKα (Thr172) (CST, #2535); anti-AMPKα (CST, #5832); anti-phospho-MEK1/2 (Ser221) (CST, #2338); anti-phospho-Erk1/2 (Thr202/Tyr204) (CST, #4370); anti-phospho-p90RSK (T359/S363) (Abcam, ab32413); anti-phospho-LKB1 (Ser428) (Abcam, ab63473); anti-phospho-Raptor (Ser792) (CST, #2083); anti-phospho-ULK1 (Ser555) (CST, #5869); anti-phospho-ULK1 (Ser757) (CST, #6888); anti-ULK1 (CST, #8054).

### Cell lines and cell culture

Human CRC cell lines HT29, RKO, and Caco2 were obtained from ATCC (Manassas, VA), and melanoma cell line A375 was given from Dr. Kikuchi (Division of Gene Therapy Science, Graduate School of Medicine, Osaka University). All cell lines were cultured with Dulbecco’s modified Eagle medium (DMEM, Sigma-Aldrich) supplemented 10% fetal bovine serum (FBS, Thermo Fisher Scientific), 100 U/mL penicillin and grown at 37 °C in a humidified atmosphere of 95% air, 5% CO_2_. BRAF inhibitors were dissolved in dimethyl-sulphoxide (DMSO) and were diluted with medium before use. Final concentration of DMSO was 0.1%.

### Small interfering RNA and transfection

The oligonucleotide small interfering RNA (siRNA) specifically targeting Atg13 (Custom Select, #:4390827) and negative control siRNA (Silence Select, #:4390843) were purchased from Life Technologies. Atg7 siRNA (SI02655373) was purchased from QIAGEN. Target sequences were as follows; sense Atg13, GAGUUUGGAUAUACCCUUUtt, antisense Atg13: AAAGGGUAUAUCCAAACUCgt; sense Atg7, CAGUGGAUCUAAAUCUCAATT, antisense Atg7, UUGAGAUUUAGAUCCACUGAT. Cells were transfected with control siRNA, Atg13 siRNA, or Atg7 siRNA using Lipofectamine RNAiMax (Life Technologies) according to the manufacturer’s instructions. After incubation of 48 hours, the medium was changed to DMEM containing selective BRAF inhibitors or DMSO. We determined the efficiency of siRNA-mediated protein knockdown by Western blot. For the BRAF inhibitors sensitivity, cells were seeded in 96-well plates at a density of 2,000 cells 100 μL per well and transfected with 10 μM of either control siRNA, Atg13 siRNA, or Atg7 siRNA. The cytotoxicity of selective BRAF inhibitors was determined by MTT and apoptosis assays.

### Cell viability and combination index (CI)

Cell viability was determined by MTT assay. Cells were seeded into 96-well plates at a density of 5,000 cells 100 μL per well and treated with selective BRAF inhibitors, CQ or their combination. After treatment, 10 μL of the cell-counting solution (Cell counting kit-8, Dojindo Molecular Technologies) was added to each well and incubated in a humidified 5% CO_2_ atmosphere at 37 °C for 2 h. The absorbance at 450 nm was determined using iMark^TM^ microplate absorbance reader (Bio-Rad Laboratories) to study the proliferation.

Drug interactions and dose-effect relationships were analyzed according to the reported method^17^,^18^, with minor modifications. For drug combinations, the combination index (CI) was calculated by the classic isobologram equation. The previous studies recommend the use of cytotoxic agents at IC_50_, but this strategy would not have been feasible in the case of RKO cells. Thus we determined CI at 50% cell death in HT29 cells and at 30% cell death in RKO cells. The CI was defined as follows: CI = [(D)_A/A+B_/(D)_A_/(D)_A_]+[(D)_B/A+B_/(D)_B_/(D)_B_] where (D)_A_ and (D)_B_ is the concentrations of drug A or B alone giving a 30% or 50% reduction in cell viability compared to a control. (D)_A+B_ is the concentration of drug A and B in combination producing a 30% or 50% reduction in cell viability compared to a control. The IC_30 or 50_ values were calculated for monotherapy. The ratio for combination of reagents was determined on the basis of IC_30 or 50_ values. We used a cut off for the CI of 0.8. We identified a synergistic effect when CIs were smaller than 0.8; an antagonistic effect when they were greater than 1.2; and an additive effect when they were between 0.8 and 1.2.

### Western blot analysis

Cell lysates was harvested from the cell lines using radio immunoprecipitation assay (RIPA) buffer (Thermo Fisher Scientific) with protease inhibitors, phosphatase inhibitors and EDTA (Thermo Fisher Scientific). Aliquots of protein were electrophoresed on SDS/PAGE Tris·HCl gels (Bio-Rad Laboratories). The proteins were separated by electrophoresis prior to transfer to PVDF membranes (Bio-Rad Laboratories) and incubated with primary antibodies overnight at 4 °C, followed by incubation with HRP-linked anti-rabbit or anti-mouse IgG (GE Healthcare Biosciences) at a dilution of 1:100,000 for 1 h at room temperature. The antigen–antibody complex was detected with the ECL Prime Western Blotting Detection Kit (GE Healthcare Biosciences). The intensity of the blots was quantified by densitometry analysis using Image lab software version 5.0 (Bio-Rad Laboratories).

### Apoptosis assay

Apoptotic rate was determined by flow cytometry with the Annexin V-fluorescein isothiocyanate (FITC) apoptosis detection kit (Biovision). Briefly, cells were collected by accutase and suspended with 500 mL of 1× binding buffer and then treated with 5 μL of Annexin V–FITC and 2.5 μL PI. After incubation of 10 minutes on ice, each sample was analyzed immediately using the BD FACSAria™ IIu instrument (BD Bioscience). We defined apoptotic cells as follow: unstained cells were classified as “live”; cells stained by Annexin V only were “early apoptotic”; cells stained by both Annexin V and PI were “late apoptotic”; and cells stained by PI only were “dead” cells.

### Immunocytochemistry for LC3 localization

Cells were seeded in 35-mm diameter ibidi dishes. After treatment of 24 h, cells were washed with 1 × PBS and fixed with 4% paraformaldehyde for 15 minutes at room temperature. Subsequently, cells were blocked by blocking-one for 1 h at room temperature, and then incubated overnight with primary antibodies, anti-LC3 antibody (1:400 diluted in PBS with 0.1% Tween). Cells were washed with 1 × BSA buffer, followed by incubation with secondary antibodies, Alexa Fluor 488 conjugated anti-rabbit antibody (1:1000 diluted in PBS with 0.1% Tween), for 1 h at room temperature. Cells were washed with 1 × PBS and then mounted with DAPI (Life technologies). Cells were visualized using a confocal laser scanning microscope (Olympus FluoView FV1000) at an objective of ×200.

### Xenograft models

Animal studies were conducted in strict accordance with the principles and procedures approved by the Committee on the Ethics of Animal Experiments of Osaka University. For xenograft models, 7-week-old female mice (BALB/c–nu/nu) were purchased from CLEA Japan, Inc. HT29 (5.0 × 10^6^) cells in a total volume of 200 μL DMEM/Matrigel (4:1 (v/v) suspension) were injected subcutaneous into the flanks. When the diameter of the subcutaneous tumor reached about 100 mm^3^, tumorbearing animals were randomly assigned to four groups that were administered Control (Vehicle plus PBS), CQ alone, PLX4032 alone, or Combination (PLX4032 plus CQ). Vehicle solution contained 5% DMSO and 1% methyl cellulose. PLX4032 was formulated in 5% DMSO, 1% methyl cellulose, and dosed at 50 mg/kg twice daily by oral gavage. CQ was dissolved in PBS and intraperitoneally administered daily at the dose of 60 mg/kg/day. Tumor volume was measured with calipers and calculated using the formula V = (ab^2^)/2, where a is the largest diameter and b is the smallest diameter. Mice were sacrificed 24 hours after the last treatment. The tumors were weighed and tumor lysates were subjected to Western blot analysis.

### Statistical analysis

Data were collected from at least three independent experiments. Data are presented as mean ± SD. Comparisons between two groups were performed by unpaired t test using JMP pro version 11 (SAS Institute, Cary, N.C., USA). A *P* value < 0.05 was considered significant.

## Results

### Autophagy is induced in BRAF^V600E^ CRC cells after treatment with selective BRAF inhibitors

**Figure 1:**
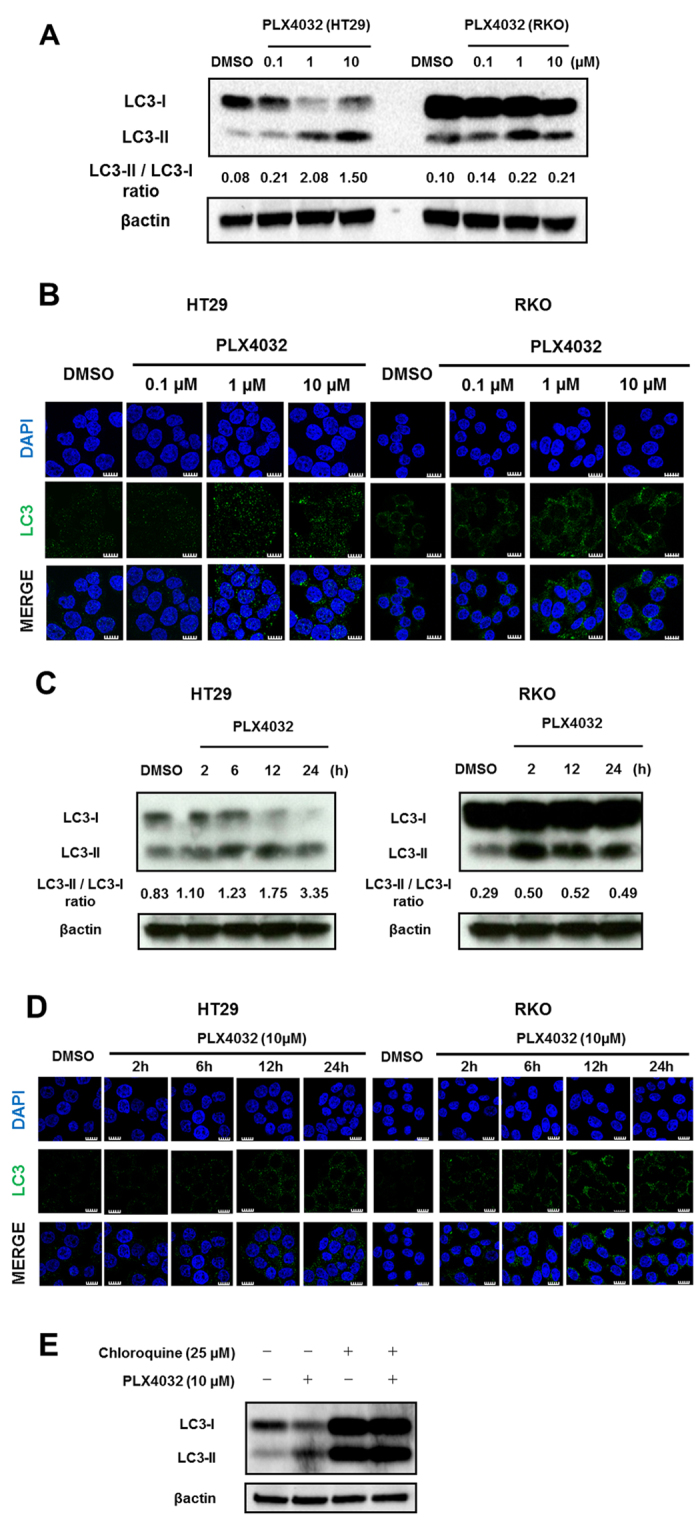
Selective BRAF inhibitors induce autophagy in BRAF^V600E^ CRC cells. (**A**) HT29 and RKO cells were treated with DMSO and 0.1, 1, or 10 μM PLX4032 for 24 h. LC3-II/LC3-I ratios were determined using Image lab software version 5.0 (Bio-Rad Laboratories). (**B**) HT29 and RKO cells were treated with DMSO and 0.1, 1, or 10 μM PLX4032 for 24 h. Cells were visualized using a confocal laser microscope (Olympus FluoView FV1000; objective, ×200). Green dots correspond with accumulated LC3 in autophagosomes and nuclei are stained with DAPI (blue). Scale bars indicate 10μm. (**C**) HT29 and RKO cells were treated with DMSO for 24 h and 10 μM PLX4032 for 2, 6, 12, and 24 h and LC3-II expression and LC3-II/LC3-I ratios were determined using Western blot analyses. (**D**) HT29 and RKO cells were treated with DMSO for 24 h and 10 μM PLX4032 for 2, 6, 12, and 24 h. Cells were visualized using a confocal laser microscope (Olympus FluoView FV1000; objective,×200). Green dots correspond with accumulated LC3 in autophagosomes and nuclei are stained with DAPI (blue). Scale bars indicate 10μm. (**E**) HT29 cells were treated with or without 10 μM PLX4032 in the presence or absence of 25 μM CQ for 24 h, and Western blot analyses of LC3 turnover were performed.

To examine the effects of selective BRAF inhibitors on autophagy, we monitored the induction of autophagy in BRAF^V600E^ HT29 and RKO CRC cells after treatment with selective BRAF inhibitors. Initially, we assessed the expression of light chain 3 (LC3), a mammalian homolog of yeast Atg8, using western blotting. During autophagy, the cytoplasmic form of LC3-I is converted to the membrane-bound lipidated form LC3-II, as detected by electrophoretic mobility shifts^8^. On the basis of a previous study^13^, A375 melanoma cells were used as a positive control (Supplementary Fig. 1A,B left panel), and dose dependency of this response was examined in HT29 cells in the presence of drugs at 0.1, 1, and 10 μM. Although LC3-II expression was not clearly increased in the presence of 0.1 μM PLX4032, the expression of LC3-I was decreased, indicating the modest activation of autophagosome formation. Accordingly, LC3-II and LC3-II/LC3-I ratios were clearly increased at 1 and 10 μM PLX4032 or PLX4720 (Fig. 1A and Supplementary Fig. 1C left panel), and in complimentary experiments, changes in autophagic activity were visually examined using confocal microscopy. In particular, PLX4032-induced punctate staining of LC3-II was increased in HT29 cells (Fig. 1B left panel), and LC3 conversion was observed as early as 2–6 h after treatment with 10 μM PLX4032 and gradually increased until 24 h (Fig. 1C,D left panel). In further experiments, 1 and 10 μM PLX4032 or PLX4720 increased LC3-II expression and LC3-II/LC3-I ratios in RKO cells (Fig. 1A,B right panel and Supplementary Fig. 1C right panel). Moreover, at 10 μM PLX4032, increased LC3-II expression was significant at 2 h after the initiation of treatment and was stable until 24 h in RKO cells (Fig. 1C,D right panel). These data indicate that LC3-II accumulates dose and time dependently in CRC cell lines. In contrast, no significant changes in LC3-II expression were observed after identical treatments in BRAF^wild-type^ Caco2 cells (Supplementary Fig. 1A, B right panel). Subsequently, to clarify whether accumulation of autophagosomes following treatments with selective BRAF inhibitors reflects induction of autophagosome formation or inhibition of autophagosome degradation, BRAF inhibitor-treated cells were exposed to chloroquine (CQ). In these experiments, accumulation of LC3-II was further enhanced, indicating that the accumulation of LC3-II in the presence of selective BRAF inhibitors follows the activation of autophagosome formation rather than the inhibition of autophagosome degradation (Fig. 1E).

### Inhibition of selective BRAF inhibitor-induced autophagy by pharmacological or siRNA inhibitors sensitizes BRAF^V600E^ CRC cells to selective BRAF inhibitors

**Figure 2:**
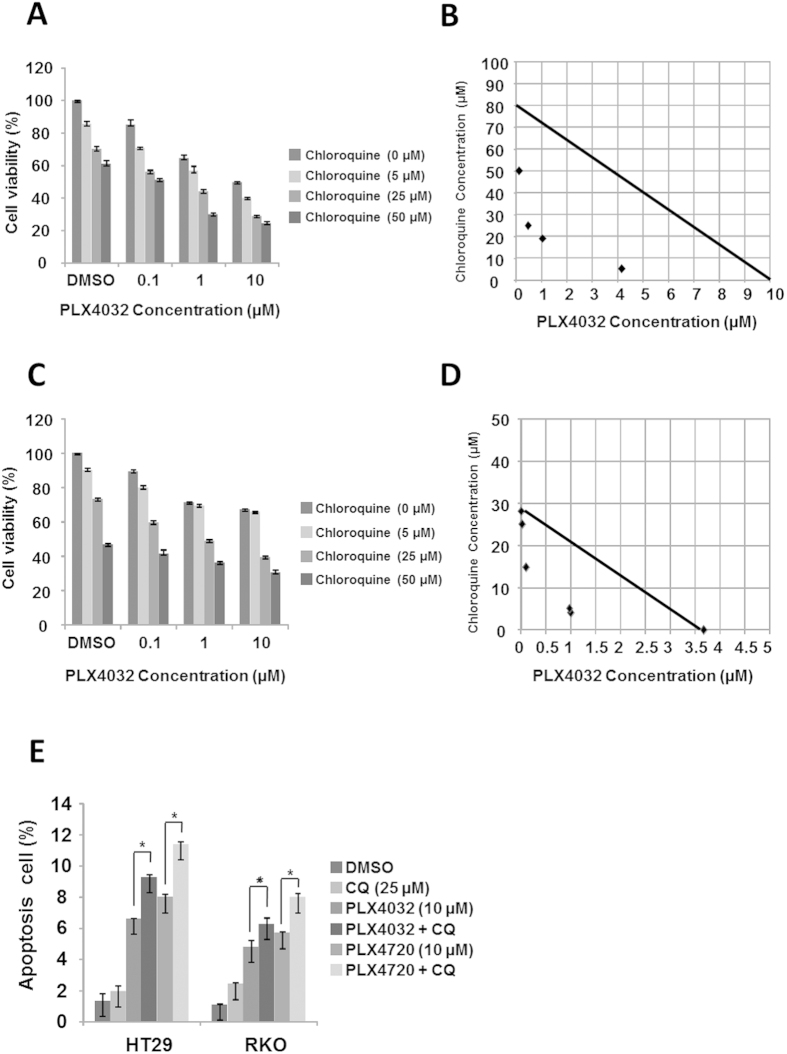
Pharmacological inhibition of autophagy by CQ increases sensitivity of BRAF^V600E^ CRC cells to PLX4032. (**A**) HT29 cells were treated with or without 0.1, 1, or 10 μM PLX4032 in the presence or absence of 0, 5, 25, or 50 μM CQ for 24 h. Cell viability was determined using MTT assays. (**B**) Isobologram analysis of interactions between PLX4032 and CQ in HT29 cells. (**C**) RKO cells were treated with or without 0.1, 1, or 10 μM PLX4032 in the presence or absence of 0, 5, 25, or 50 μM CQ for 24 h. Cell viability was determined using MTT assays. (**D**) Isobologram analysis of interactions between PLX4032 and CQ in RKO cells. (**E**) HT29 and RKO cells were treated with PLX4032 or PLX4720 at 10 μM in the presence or absence of CQ at 25 μM for 24 h. Apoptosis rates were determined using flow cytometry and cells were defined as early or late apoptotic cells. Data are presented as means and standard deviations of at least three independent experiments; *p < 0.05.

To elucidate the role of selective BRAF inhibitor-induced autophagy, autophagy was inhibited using the pharmacological inhibitor CQ or using RNA interference of Atg13 or Atg7. After exposure of cells to PLX4032 and CQ, which blocks downstream autophagy pathways, drug interactions and dose-effect relationships were assessed using a CI according to the methods of Chou and Talalay^17^,^18^. Initially, the effects of monotherapy were examined in HT29 cells at CQ concentrations of 0, 0.01, 0.1, 1, 10, 100, and 1000 μM, and whereas 1 μM CQ had almost no inhibitory effect after 24 h, CQ concentrations of more than 1 μM inhibited proliferation in a dose-dependent manner (Supplementary Fig. 2A). Subsequently, combined treatments of HT29 cells resulted in improved cytotoxic effects at 24 h for every regimen compared with respective monotherapies (Fig. 2A). Moreover, analysis of combined effects revealed synergy with a CI value of 0.523 ± 0.15 (Fig. 2B). In subsequent experiments using RKO and A375 cells, combination treatments improved the cytotoxic effects of all monotherapeutic regimens at 24 h (Fig. 2C and Supplementary Fig. 2B,C), and analysis of combined effects revealed synergy with CI values of 0.577 ± 0.19 and 0.505 ± 0.15, respectively (Fig. 2D and Supplementary Fig. 2D). In further experiments, the extent of apoptosis was investigated at 24 h after single and combination treatments with PLX4032 or PLX4720 and CQ using apoptosis assays. These experiments revealed significantly higher percentages of early and late apoptotic cells after combination treatments in all cell lines (Fig. 2E and Supplementary Fig. 2E).

**Figure 3:**
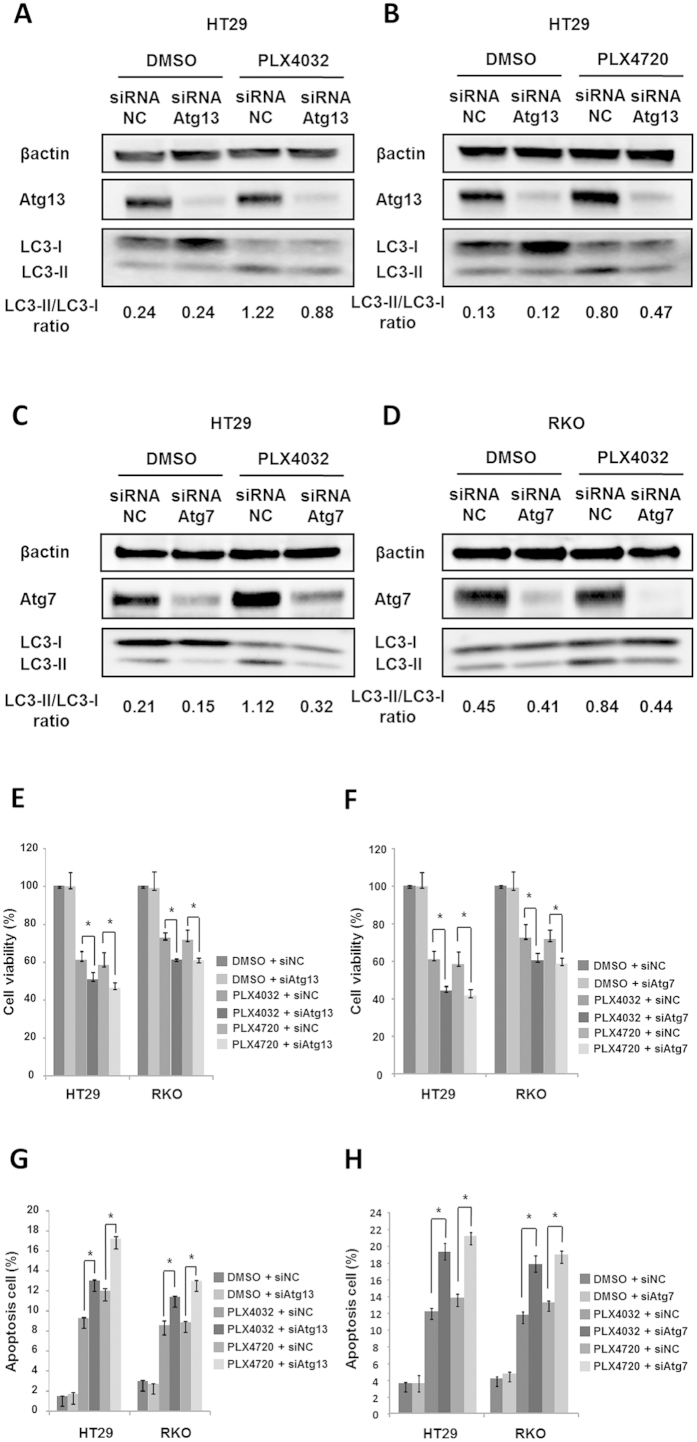
Inhibition of autophagy by Atg13 or Atg7 siRNA increases sensitivity of BRAF^V600E^ CRC cells to PLX4032. (**A,B**) HT29 cells were transfected with Atg13 siRNA or control siRNA and were then treated with DMSO and PLX4032 or PLX4720 at 10 μM for 24 h. LC3 and Atg13 expression was determined using western blot analyses. (**C,D**) HT29 and RKO cells were transfected with Atg7 siRNA or control siRNA and were then treated with DMSO or PLX4032 at 10 μM for 24 h. LC3 and Atg7 expression was determined using western blot analyses. (**E,F**) HT29 and RKO cells were transfected with control siRNA, Atg13 siRNA, or Atg7 siRNA and were treated with DMSO, PLX4032, or PLX4720 at 10 μM for 24 h. Cell viability was then determined using MTT assays. (**G,H**) Apoptosis assay in HT29 and RKO cells transfected with control siRNA, Atg13 siRNA, or Atg7 siRNA. Apoptotic rates were determined using flow cytometry, and early and late apoptotic cells were distinguished. Data are presented as means and standard deviations of at least three independent experiments; *p < 0.05.

To elucidate associated mechanisms, we downregulated autophagy by targeting Atg13 or Atg7 which was gene to be associated with autophagy. Total protein expressions of Atg13 or Atg7 were stable in the presence of PLX4032 at all doses and times (Supplementary Fig. 3A–D) but was significantly decreased after treatment with targeted siRNA. Moreover, knockdown of Atg13 or Atg7 suppressed LC3-II expression in selective BRAF inhibitor-treated cells (Fig. 3A–D and Supplementary Fig. 3E,F), indicating that autophagy is suppressed under physiological conditions. Atg13 or Atg7 knockdown also enhanced selective BRAF inhibitor-induced apoptosis and decreased cell viability in HT29 and RKO cells (Fig. 3E–H).

### AMPK phosphorylation is required for selective BRAF inhibitor-induced autophagy

**Figure 4:**
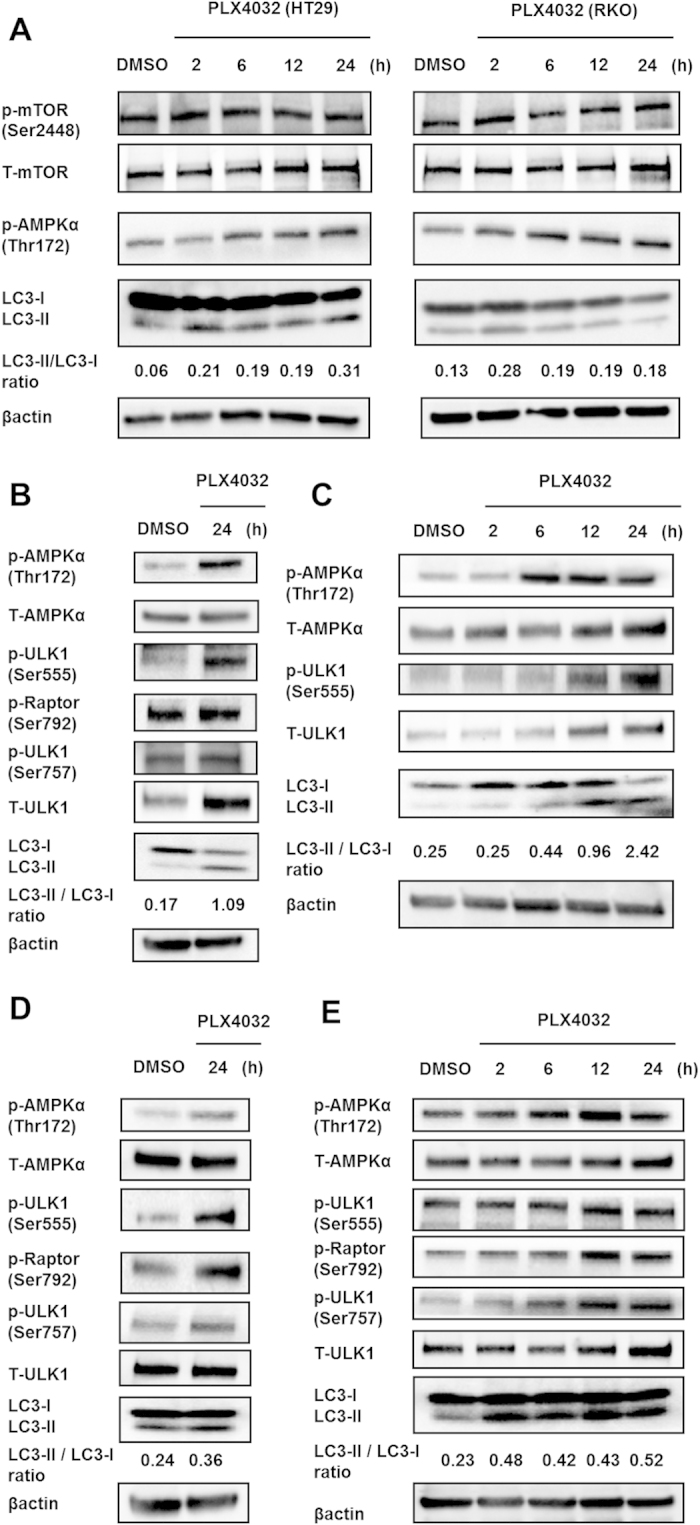
AMPK phosphorylation is required for ULK1 function during PLX4032-induced autophagy. (**A**) Western blot assays of AMPKα (Thr172) and mTOR (Ser2448) phosphorylation and LC3 expression in HT29 and RKO cells after treatment with DMSO for 24 h and 10 μM PLX4032 for 2, 6, 12, or 24 h. (**B**) Western blot analyses of phospho-AMPKα (Thr172), phospho-ULK1 (Ser555), phospho-Raptor (Ser792), phospho-ULK1 (Ser757), and LC3 in HT29 cells after treatment with DMSO and 10 μM PLX4032 for 24 h. (**C**) Western blot analyses of phospho-AMPKα (Thr172), phospho-ULK1 (Ser555), and LC3 in HT29 cells after treatment with DMSO for 24 h and 10 μM PLX4032 for 2, 6, 12, and 24 h. (**D**) Western blot analyses of phospho-AMPKα (Thr172), phospho-ULK1 (Ser555), phospho-Raptor (Ser792), phospho-ULK1 (Ser757), and LC3 in RKO cells after treatment with DMSO and 10 μM PLX4032 for 24 h. (**E**) Western blot analyses of phospho-AMPKα (Thr172), phospho-ULK1 (Ser555), phospho-Raptor (Ser792), phospho-ULK1 (Ser757), and LC3 in RKO cells after treatment with DMSO for 24h and 10 μM PLX4032 for 2, 6, 12, and 24 h.

Autophagy can be induced by alterations in various growth factor signaling pathways within the tumor microenvironment, and the major direct signaling regulators of autophagy include mTORC1, AMPK, and specific components of the ER stress response^19^. To investigate mechanisms by which selective BRAF inhibitors induce autophagy, protein expression of these molecules was determined using western blotting. Although no significant changes in phospho-mTOR (ser2448) expression were observed after treatment with PLX4032, compared with DMSO-treated cells, PLX4032 increased the phosphorylation of AMPK (Thr172) after 24 h in HT29 and RKO cells (Fig. 4A). Phosphorylation of AMPK (Thr172) was also increased in the presence of increased LC3II/LC3I ratios following treatments with inhibitors of BRAF^V600E^. In addition, AMPK activity was elevated at early time points after treatment with PLX4032, indicating that AMPK activation is associated with PLX4032-induced autophagy in BRAF^V600E^ CRC cells. Additionally, combination treatments with PLX4032 or PLX4720 and CQ increased AMPK phosphorylation (Thr172) at 24 h in HT29 cells (Supplementary Fig. 4A,B).

In further experiments, associations between AMPK and selective BRAF inhibitor-induced autophagy were investigated in HT29 and RKO cells. Several previous studies report roles of AMPK in the control of autophagy^20^ and show involvement of the mTORC1 pathway following phosphorylation of the regulatory associated protein mTOR (Raptor) and the tuberous sclerosis complex 2 (TSC2). However, whereas inhibition of mTORC1 correlates with increased autophagy, AMPK directly activates ULK1, which is a key initiator of autophagy. In present experiments, AMPK was markedly phosphorylated at approximately 6 h after treatments with inhibitors of BRAF^V600E^, and ULK1 phosphorylation and (Ser555) autophagic activity were increased at 12 h compared with DMSO-treated cells. Moreover, changes in degrees of AMPK and ULK1 phosphorylation were time dependent (Fig. 4B,C), and similar changes were observed after exposure to PLX4720 (Supplementary Fig. 5A,B). In contrast, the effects of mTOR-dependent phosphorylation on the AMPK–ULK1 interaction did not produce significant changes in Raptor (Ser792) or ULK1 (Ser757) phosphorylation after 24 h treatments of HT29 cells (Supplementary Fig. 5C). These results show that selective BRAF inhibitors induce autophagy in HT29 cells, likely through direct AMPK-ULK1 interactions. In contrast with experiments in HT29 cells, PLX4032 treatments led to increased phosphorylation of Raptor (Ser792) and ULK1 (Ser757) in RKO cells (Fig. 4D,E). These data indicate that AMPK stimulates autophagy by inhibiting mTORC1 at the level of Raptor (Ser792) in addition to stimulating direct AMPK-ULK1 interactions in RKO cells.

### Downregulation of oncogenic BRAF signaling stimulates AMPK via the downstream MEK-Erk-p90RSK kinase signaling cascade

**Figure 5:**
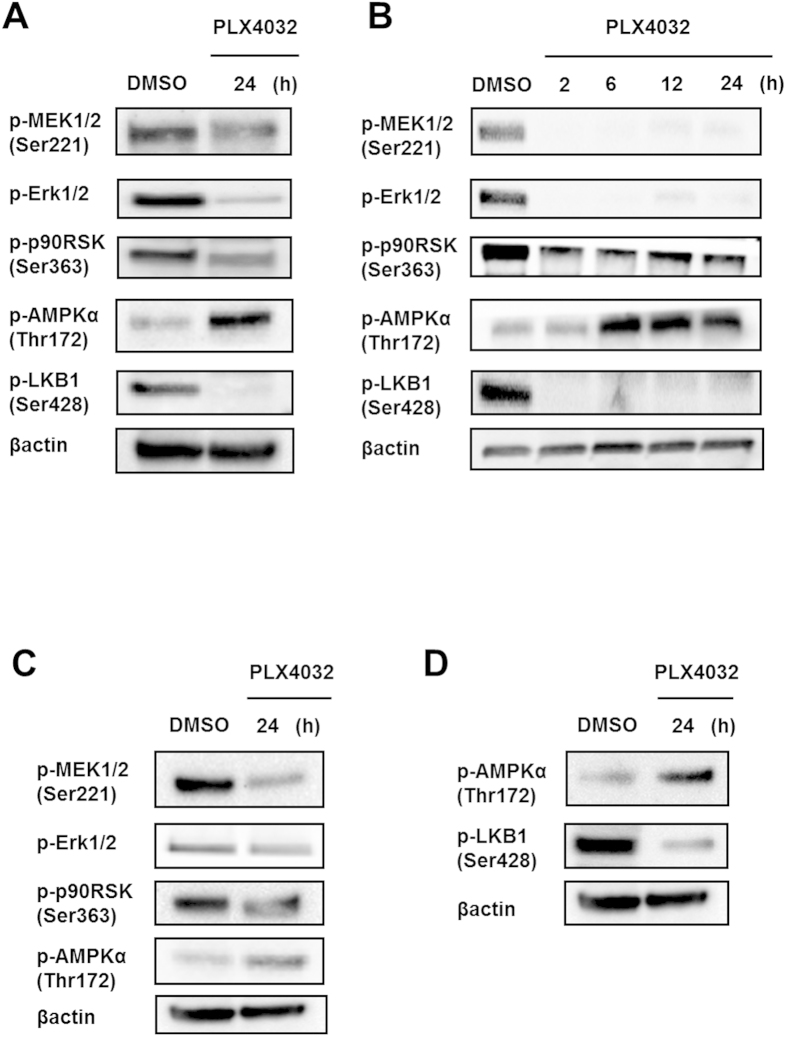
Downregulation of BRAF signaling by PLX4032 activates AMPK by inactivating LKB1 (Ser428). (**A**) Western blot analyses of phospho-MEK1/2 (Ser221), phospho-Erk1/2, phospho-p90RSK (Ser363), phospho-AMPKα (Thr172), and phospho-LKB1 (Ser428) in HT29 cells after treatment with DMSO and 10 μM PLX4032 for 24 h. (**B**) Western blot analyses of phospho-MEK1/2 (Ser221), phospho-Erk1/2, phospho-p90RSK (Ser363), phosphor-AMPKα (Thr172), and phospho-LKB1 (Ser428) in HT29 cells after treatment with DMSO for 24 h and 10 μM PLX4032 for 2, 6, 12, or 24 h. (**C**) Western blot analyses of phospho-MEK1/2 (Ser221), phospho-Erk1/2, phospho-p90RSK (Ser363), and phospho-AMPKα (Thr172) in RKO cells after treatment with DMSO and 10 μM PLX4032 for 24 h. (**D**) Western blot analyses of phospho-AMPKα (Thr172) and phospho-LKB1 (Ser428) in RKO cells after treatment with DMSO and 10 μM PLX4032 for 24 h.

Interactions between AMPK and RAF-MEK-ERK pathways have been documented in various human cancer cells, including colon cancer^15^,^16^. Accordingly, negative regulation of AMPK in BRAF^V600E^ CRC cells suggests that AMPK or its upstream kinases may be targets of the MEK-ERK-RSK signaling cascade. Thus, to evaluate the relevance of AMPK inhibition by MEK-ERK-RSK signaling, phosphorylation of MEK-Erk-p90RSK and AMPK was determined in HT29 cells. Although untreated HT29 cells showed strong MEK-Erk-p90RSK phosphorylation but no AMPK phosphorylation, PLX4032-treated HT29 cells showed strong AMPK phosphorylation and no MEK-Erk-p90RSK phosphorylation. These data suggest an inverse correlation between AMPK and MEK-Erk-p90RSK signaling pathways (Fig. 5A). Moreover, MEK-Erk-p90RSK was inactivated after only 2 h treatments and remained ineffective for 24 h, and AMPK phosphorylation was markedly increased, further suggesting inactivation of MEK-Erk-p90RSK signaling (Fig. 5B). Similar changes were also observed after treatments with PLX4720 (Supplementary Fig. 6A,B), with a corresponding negative correlation between AMPK and MEK-Erk-p90RSK phosphorylation levels in RKO cells (Fig. 5C). The present data suggest that the activity of AMPK is negatively regulated by the BRAF^V600E^ mutation, presumably via downstream MEK-Erk-p90RSK kinase signaling.

Because phosphorylation of LKB1 at Ser428 (Ser431 in mouse) by p90RSK was previously demonstrated *in vitro* and *in vivo*^21^, we determined whether the effects of selective BRAF inhibitors on AMPK activity are dependent on LKB1. In these experiments, degrees of LKB1 (Ser428) and MEK-Erk-p90RSK phosphorylation were determined in HT29 cells within 24 h exposure to PLX4032. Accordingly, downregulation of BRAF^V600E^ expression by PLX4032 led to decreased LKB1 phosphorylation at Ser428 and increased phosphorylation of AMPK at Thr172 (Fig. 5A,B). These data were corroborated by experiments in RKO cells (Fig. 5D), indicating that selective BRAF inhibitors phosphorylate AMPK by disinhibiting LKB1.

### Autophagy inhibition chloroquine enhances the anti-tumor effect of PLX4032 *in vivo*

**Figure 6:**
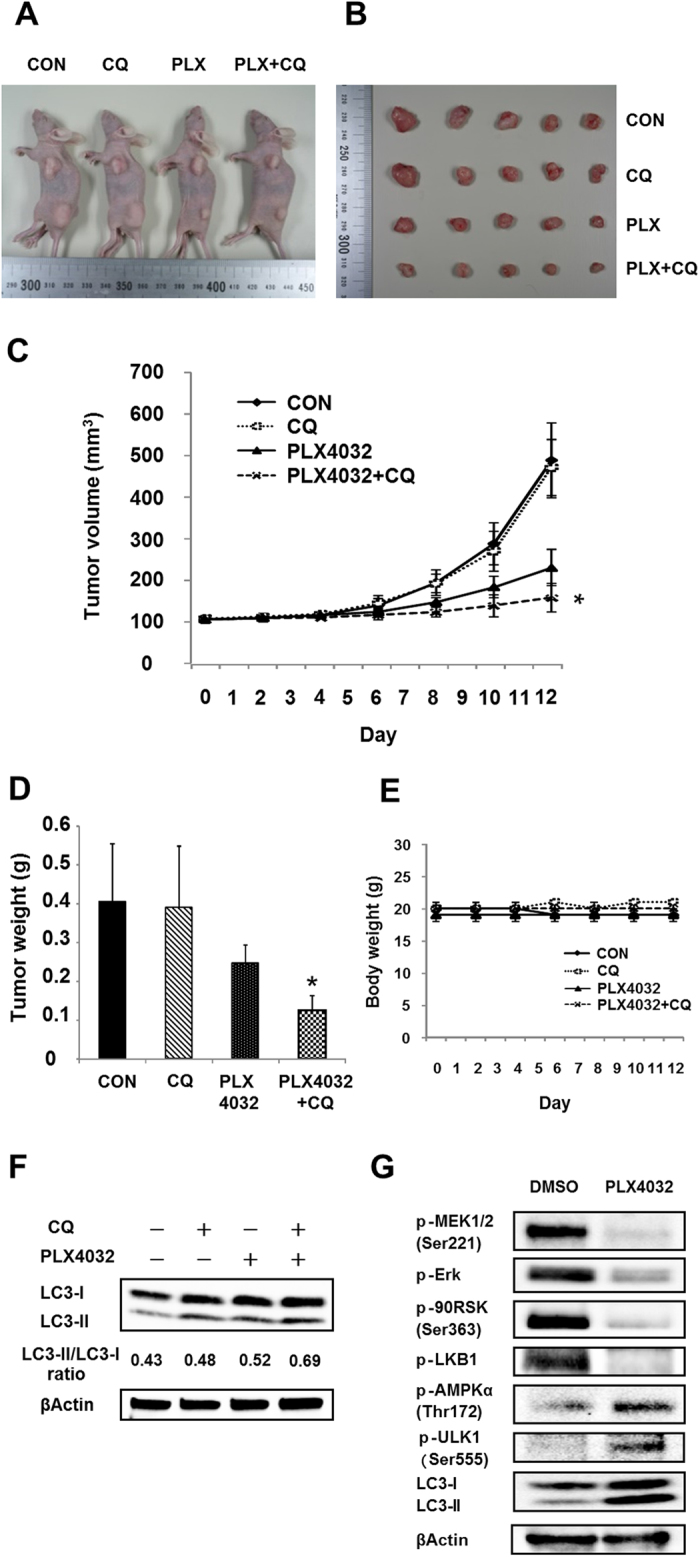
Autophagy inhibition chloroquine enhances the anti-tumor effect of PLX4032 in HT29 xenograft models. (**A**) A typical case in each group. (**B**) Tumors from nude mice in each group. (**C–E**) Tumor volume, tumor weight, and body weight in each group. (**F**) Tumor lysate was subjected to Western blot for LC3 and βActin. (**G**) Tumor lysate was subjected to immunoblotting for phospho-MEK1/2 (Ser221), phospho-Erk1/2, phospho-p90RSK (Ser363), phospho-AMPKα (Thr172), LC3, and βActin. BRAF mutant CRC xenografts derived from HT29 cells were treated with Control (vehicle plus PBS), PLX4032 alone (50 mg/kg, twice daily), CQ alone (60 mg/kg, daily), or combination therapy (PLX4032 plus CQ) for 13 day (Day0 – Day12). Average percent change in tumor volume relative to initial tumor volume is shown. Error bars represent SD. Asterisks represent p < 0.05 for combined PLX4032 plus CQ vs. all other treatment groups.

To further determine the therapeutic benefit of inhibiting autophagy in combination with PLX4032, mice with HT29 xenograft were randomly assigned to four groups when the volume of the subcutaneous tumor reached approximately 100 mm^3^: Control (CON), CQ alone, PLX4032 alone, or PLX4032 + CQ (Fig. 6A). As shown in Fig. 6B–D, CQ alone had no effect on the growth of tumors, and PLX4032 alone displayed moderate anti-tumor activity. In contrast, combination of CQ with PLX4032 significantly reduced tumor growth compared to the PLX4032 alone. Furthermore, no significant weight loss was observed by combination treatments (Fig. 6E).

We then investigated whether PLX4032 induces autophagy in HT29 xenografted tumors. Consistent with the *in vitro* results, tumors treated with PLX4032 or PLX4032 plus CQ displayed accumulation of LC3-II on western blot compared with those from vehicle-treated mice (Fig. 6F). In addition, the LC3-II/LC3-I ratio and expression of LC3-II are much higher in PLX4032 plus CQ group than that in PLX4032 or CQ alone. We next investigated the mechanism of PLX4032 induced autophagy *in vivo*. Consistence with the *in vitro* findings, PLX4032 induced autophagy through inhibition of MEK-Erk-p90RSK signaling pathways and phosphorylation of AMPK. (Fig. 6G). These findings suggest that PLX4032 induced autophagy through inhibition of MEK-Erk-p90RSK signaling pathways and phosphorylation of AMPK, and inhibition of autophagy by CQ can potently enhance the anti-tumor effect of PLX4032 *in vivo*.

## Discussion

In this study, autophagy was induced by phosphorylated AMPK in BRAF^V600E^ CRC cells after exposure to selective BRAF inhibitors and provided protection against the cytotoxic effects of selective BRAF inhibitors. Here we show three complementary lines of evidence that autophagy is induced in BRAF^V600E^ CRC cells after exposure to selective BRAF inhibitors. Subsequently, functional roles of selective BRAF inhibitor-induced autophagy were demonstrated, and molecular associations between the BRAF^V600E^ mutation and treatment-induced autophagy were elucidated. To the best of our knowledge, the cytotoxic roles of selective BRAF inhibitor-induced autophagy have not been examined in BRAF^V600E^ CRC cells.

The present experiments showed convincing evidence that autophagy is induced after exposure of BRAF^V600E^ CRC cells to selective BRAF inhibitors. In particular, LC3 is widely used to monitor autophagy, and LC3-II expression usually correlates closely with numbers of autophagosomes. Hence, assessments of autophagosome numbers were performed using light microscopic and biochemical methods as previously described^8^ and showed dose-dependent increases in punctate LC3 staining. Moreover, LC3-I conversion to LC3-II was increased, resulting in the accumulation of LC3-II after exposure of cells to selective BRAF inhibitors. However, accumulations of autophagosomes are not always indicative of increased autophagy and may alternatively reflect blockade of autophagosomal maturation and completion of the autophagy pathway^8^. Hence, LC3 turnover assays were performed in the presence of CQ and showed evidence that selective BRAF inhibitors increase numbers of autophagosomes and activate autophagic flux.

In subsequent experiments, we investigated functional roles of selective BRAF inhibitor-induced autophagy using CQ as a specific pharmacological inhibitor of autophagy. CQ has been widely used for many years without significant toxicity in malaria prophylaxis and has been implicated as a potential adjunct to anticancer treatments. In the present study, CQ inhibited autophagy, increased numbers of apoptotic cells, and decreased cell viability. In accordance with the requirement of Atg13 in complex with ULK1 and FIP200 for initiation of autophagy^22^, specific siRNA against Atg13 increased cell death markedly as treatments with CQ. Similar changes were also observed after treatment with targeted Atg7, which is an autophagy-related gene. Autophagy is known to serve as a cytoprotective mechanism that allows escape from anticancer treatment-induced cell death and can compromise the efficacy of therapeutic interventions in cancer cells^23^. The present data suggest that both initial inhibition of autophagy by Atg13 or Atg7 knockdown and subsequent inhibition by CQ sensitizes BRAF^V600E^ CRC cells by converting the autophagic process into an apoptotic process. To further determine the therapeutic benefit of inhibiting autophagy in combination with PLX4032, we examined whether selective BRAF inhibitor-induced autophagy can be therapeutically targeted using appropriate xenograft models. In HT29 xenograft models, although PLX4032 monotherapy was shown to be superior to that of CON or CQ monotherapy, greater efficacy was achieved with combination therapy. Furthermore, there were no adverse effects detected in mice treated with PLX4032 plus CQ. Thus, whereas autophagy can protect cancer cells from cytotoxic treatments, its inhibition can improve the effects of selective BRAF inhibitors in BRAF^V600E^ CRC cells.

**Figure 7:**
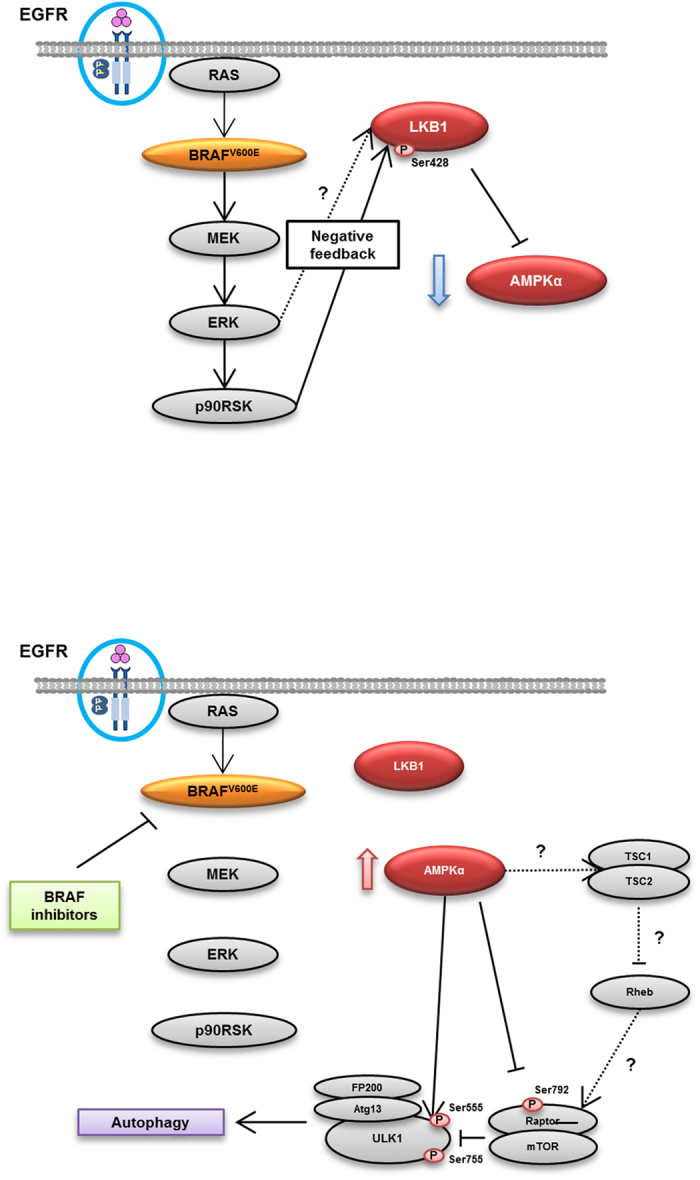
A model of a molecular linkage between the BRAF^V600E^ mutant protein, selective BRAF inhibitor-induced autophagy, and AMPK phosphorylation.

Finally, AMPK activation was central to BRAF inhibitor-induced autophagy, indicating a mechanistic linkage that may be exploited in the treatment of inhibitor resistant BRAF^V600E^ CRC cells. AMPK acts as a cellular sensor for energy balance status, and reports showing that AMPK activation suppresses cell growth and proliferation have warranted speculation that AMPK contributes to tumor suppressor pathways under stress conditions^24^,^25^,^26^. However, several reports associate AMPK with autophagy^27^,^28^,^29^,^30^ and corresponding protection against cytotoxic treatments, creating controversy about the role of AMPK in BRAF^V600E^ CRC. In the present experiments, exposure to selective BRAF inhibitors activated AMPK in conjunction with clearly increased autophagic activity, and subsequent inhibition of autophagy sensitized BRAF^V600E^ CRC cells to selective BRAF inhibitors. Hence, AMPK likely facilitates escape from cell death following treatments with selective BRAF inhibitors as an inducer of autophagy during the early phase. However, whereas direct interactions of the AMPK-ULK1 signaling pathway were observed in HT29 and RKO cells, selective BRAF inhibitors also induced autophagy by inhibiting mTORC1 at the level of Raptor (Ser792) in RKO cells. Moreover, subsequent experiments showed that AMPK expression is relatively low in BRAF^V600E^ cells, potentially reflecting relative MEK-ERK-RSK kinase signaling activities in these cells. In recent studies, reduced AMPK levels were shown in some cases of hepatocellular carcinomas^31^, and BRAF^V600E^ was shown to regulate AMPK signaling by inhibiting Lkb1/Stk11 in melanoma cells^15^,^16^. Moreover, LKB1 was found to be the key upstream kinase for activation of AMPK^32^. Accordingly, the present data show an inverse correlation between the activities of MEK-ERK-RSK signaling and AMPK in BRAF^V600E^ CRC cells, suggesting that BRAF mutations activate phospho-MEK, Erk, and p90RSK without activating AMPK (Fig. 7 upper panel) and that inhibition of BRAF using selective BRAF inhibitors leads to inactivation of phospho-MEK, Erk, and p90RSK and activation of AMPK (Fig. 7 lower panel). Intriguingly, the present data also suggested that activation of AMPK by the BRAF^V600E^ mutant protein is impaired due to phosphorylation of LKB1, and loss of LKB1 activity following inhibition of BRAF led to activation of AMPK (Fig. 7). Although these observations require confirmation in further studies of the roles of LKB1 and AMPK, consequent improvements in understanding of selective BRAF inhibitor-induced autophagy may lead to improved therapeutic strategies for patients with BRAF^V600E^ CRC.

In summary, selective BRAF inhibitors induced autophagy in BRAF^V600E^ CRC cells. Inhibition of autophagy protected cancer cells from the cytotoxic effects of selective BRAF inhibitors, in part, by promoting apoptosis in the presence of selective BRAF inhibitors. Critically, the present observations demonstrate that AMPK activity is sensitive to MEK-Erk-p90RSK signaling in BRAF^V600E^ CRC cells. Further clarification of the mechanisms associated with this molecular linkage in BRAF^V600E^ CRC cells may facilitate the development of novel combination therapies with improved efficacy for patients with BRAF^V600E^ CRC.

## Additional Information

**How to cite this article**: Sueda, T. *et al.* BRAF^V600E^ inhibition stimulates AMP-activated protein kinase-mediated autophagy in colorectal cancer cells. *Sci. Rep.* **6**, 18949; doi: 10.1038/srep18949 (2016).

## Supplementary Material

Supplementary Information
