# Supplementary material for: BRAFV600E inhibition stimulates AMP-activated protein kinase-mediated autophagy in colorectal cancer cells
Source: Sci Rep. 2016 Jan 11;6:18949. doi: 10.1038/srep18949 (PMC4707439; doi:10.1038/srep18949)
Supplement: Supplementary Information [file srep18949-s1.doc]

**Title:**

BRAF^V600E^ inhibition stimulates AMP-activated protein kinase-mediated autophagy in colorectal cancer cells

**Authors:**

Toshinori Sueda^1,2*^, Daisuke Sakai^1*^, Koichi Kawamoto^2*^, Masamitsu Konno^2^, Naohiro Nishida^1^, Jun Koseki^3^, Hugh Colvin^1,2,3^, Hidekazu Takahashi^2^, Naotsugu Haraguchi^2^, Junichi Nishimura^2^, Taishi Hata^2^, Ichiro Takemasa^2^, Tsunekazu Mizushima^2^, Hirofumi Yamamoto^4^, Taroh Satoh^1^, Yuichiro Doki^1,2,3^, Masaki Mori^1,2,3**^, Hideshi Ishii^1,3**^

**Authors’ affiliations:**

^1^ Department of Frontier Science for Cancer and Chemotherapy, Osaka University, Graduate School of Medicine, 2-2, Yamadaoka, Suita, Osaka, 565-0871, Japan

^2^ Department of Gastrointestinal Surgery, Osaka University, Graduate School of Medicine, 2-2, Yamadaoka, Suita, Osaka, 565-0871, Japan

^3^ Department of Cancer Profiling Discovery, Osaka University, Graduate School of Medicine, 2-2, Yamadaoka, Suita, Osaka, 565-0871, Japan

^4^ Department of Molecular Pathology, Osaka University Graduate School of Medicine and Health Science, 1-7, Yamadaoka, Suita, Osaka, 565-0871, Japan

**Supplementary materials**


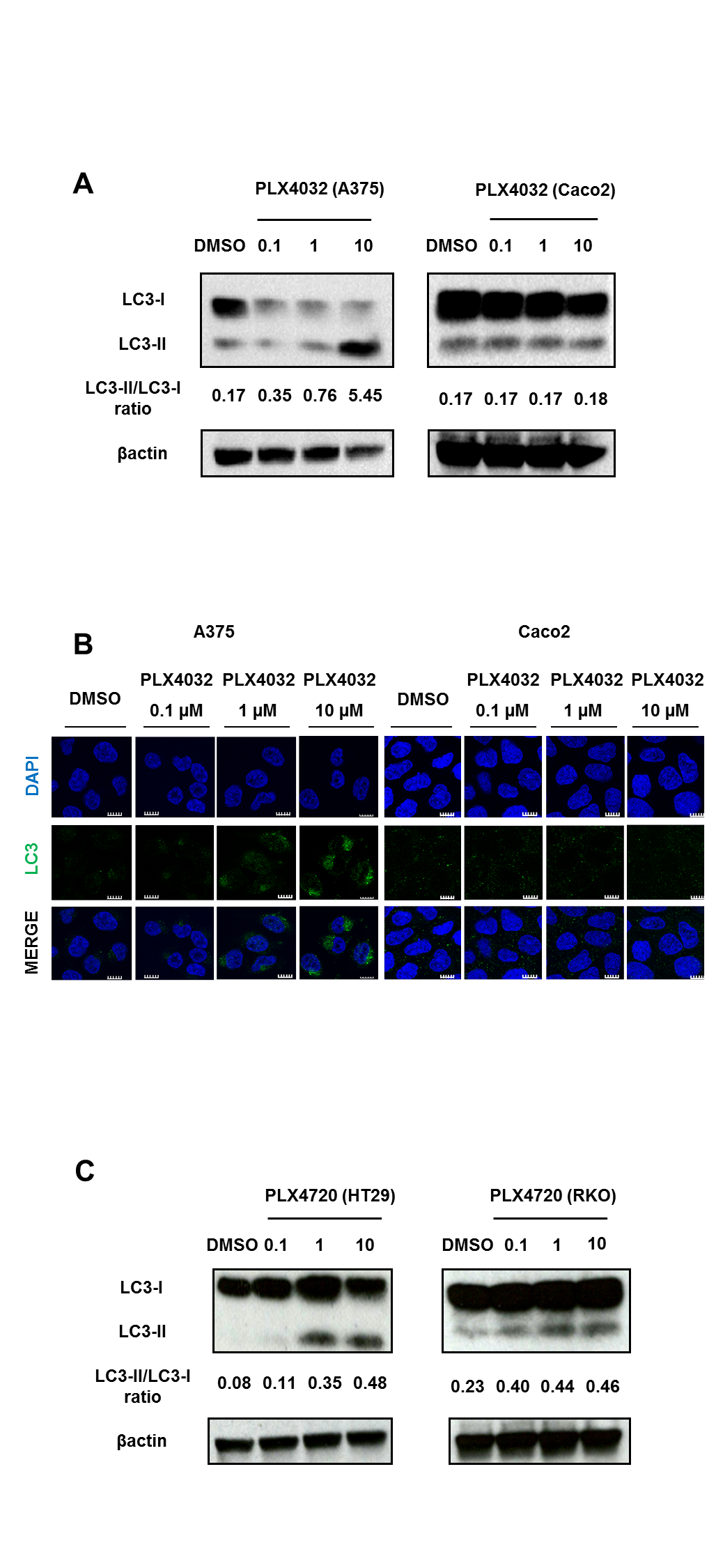


**Supplementary Figure 1. Selective BRAF inhibitor PLX4720 induces autophagy in BRAF^V600E^ CRC cells.**

(A) Western blot assays of LC3 in A375 melanoma cells and Caco2 CRC cells after treatment with DMSO or PLX4032 at 10 µM for 24 h. A375 melanoma cells used are positive control, whereas Caco2 cells are negative control of autophagy. (B) A375 Melanoma cells and Caco2 CRC cells were treated with DMSO and 0.1, 1, or 10 µM PLX4032 for 24 h. Cells were visualized using a confocal laser microscope (Olympus FluoView FV1000; objective, × 200). Green dots correspond with accumulated LC3 in autophagosomes and nuclei are stained with DAPI (blue). Scar bars indicate 10 µm. (C) Western blot assays of LC3 in HT29 and RKO cells after treatment with DMSO or PLX4720 at 0.1, 1, or 10 µM for 24 h.


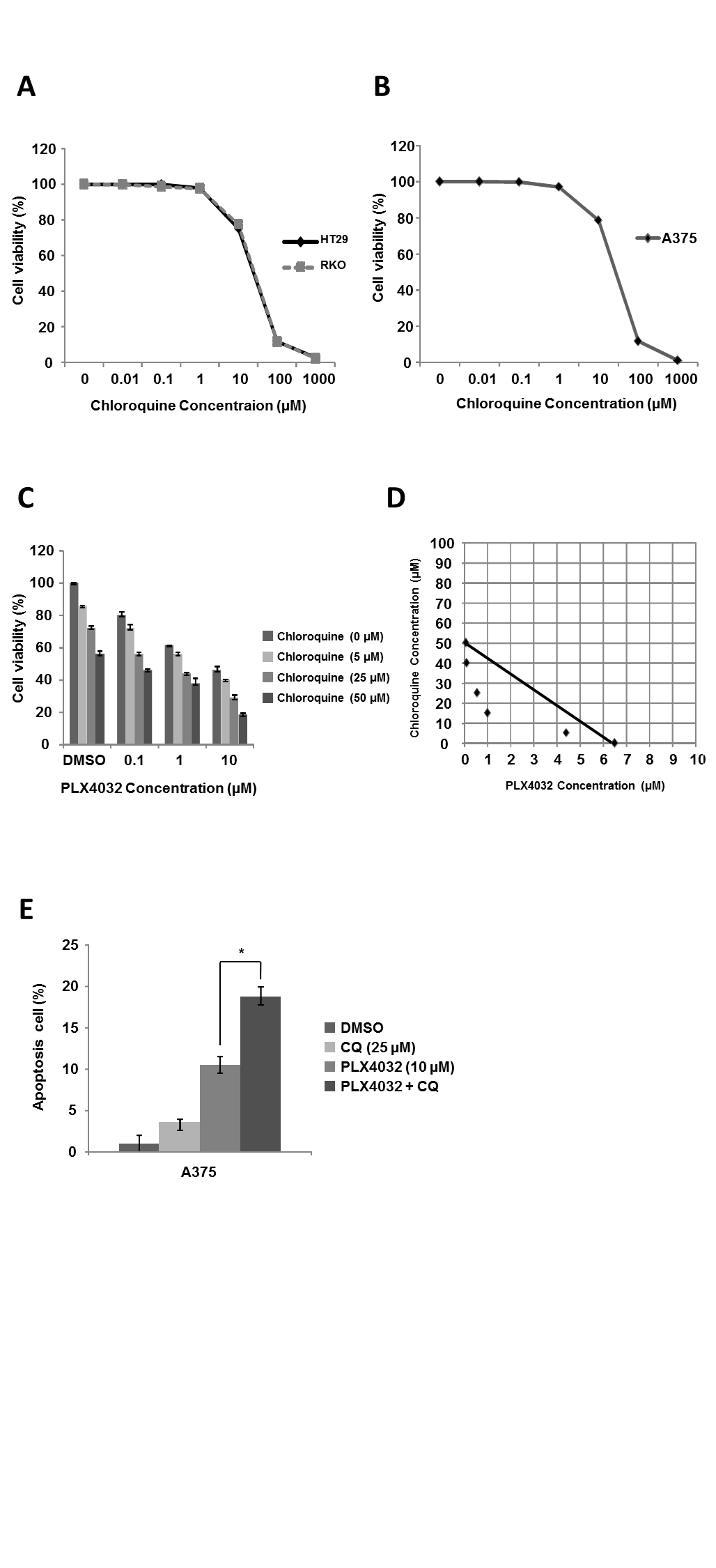


**Supplementary Figure 2. Pharmacological inhibition of autophagy by CQ sensitized A375 melanoma cells to PLX4032.**

(A, B) HT29, RKO and A375 cells were treated with CQ at 0, 0.01, 0.1, 1. 10, 100, or 1000 µM for 24 h. Cell viability was measured by MTT assay. (C) HT29 cells were treated with or without PLX4032 at 0.1, 1, or 10 µM in the presence or absence of CQ at 0, 5, 25, or 50 µM for 24 h. Cell viability was measured by MTT assay. (D) Isobologram analysis of interaction between PLX4032 and CQ in HT29 cells. CI was defined as follow; CI = [(D)_A/A+B_/(D)_A_/(D) _A_] + [(D)_B/A+B_/(D)_B_/(D)_B_]. (E) A375 cells were treated with PLX4032 at 10 μM in the presence or absence of CQ at 25 µM for 24 h. Apoptotic rates were determined by flow cytometry. Apoptosis cells include both early and late apoptosis cells. The error bars represent the S.D. of three independent replicates. Column values = average of at least three independent experiments; error bars represent standard deviation from the mean of triplicate experiments.

**
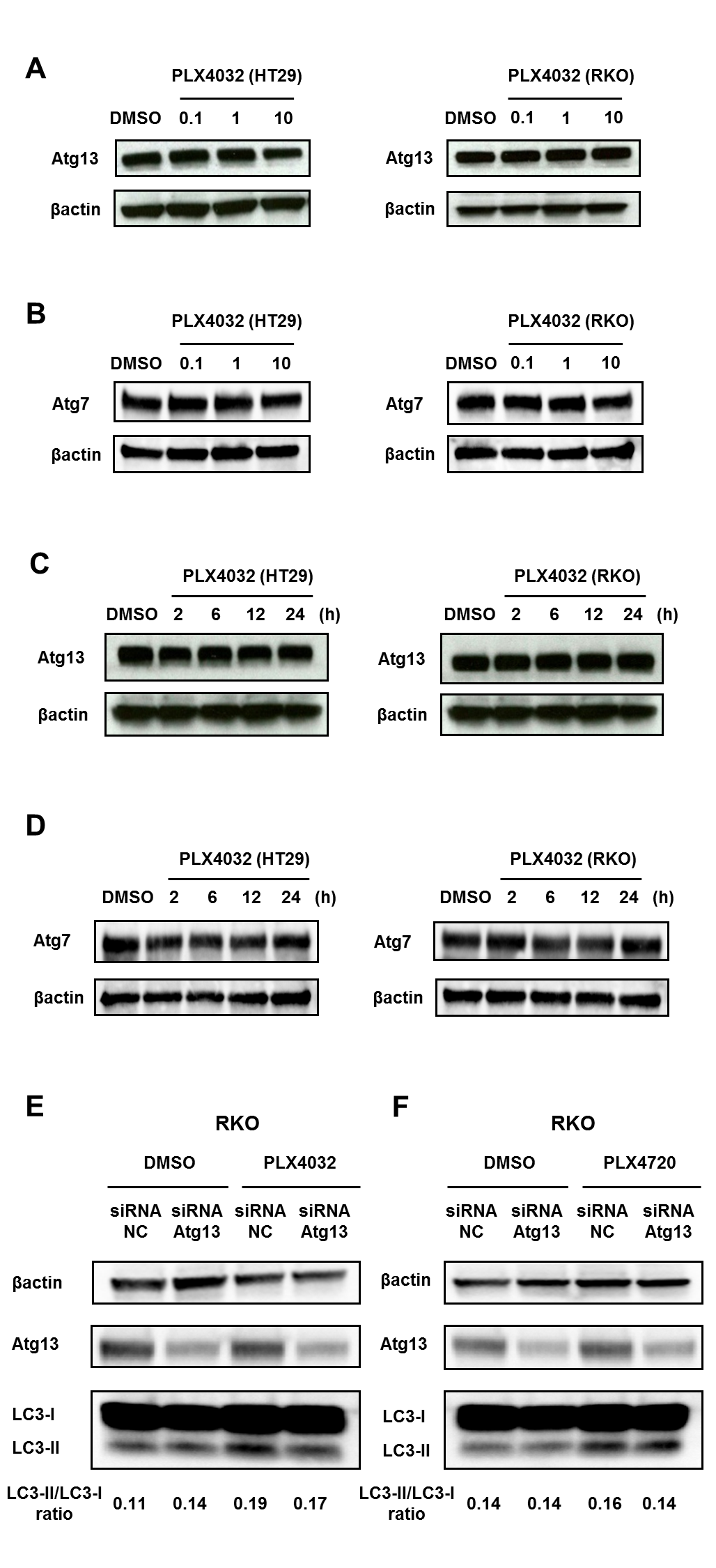
**

**Supplementary Figure 3.** Knockdown of Atg13 suppressed LC3-II expression in selective BRAF inhibitors-treated RKO cells**.**

(A,B) HT29 and RKO cells were treated with DMSO or PLX4032 at 0.1, 1, or 10 µM for 24 h. (C,D) HT29 and RKO cells were treated with DMSO for 24 h or PLX4032 at 10 µM for 2, 6, 12, or 24 h. Total protein amount of Atg13 or Atg7 was consistent over treatment with PLX4032 in a dose and time-course manner. (D, E) RKO cells were transfected with Atg13 siRNA or control siRNA, and then treated with DMSO, PLX4032, or PLX4720 at 10 µM for 24 h. LC3 and Atg13 expression was determined using western blot analyses.

**
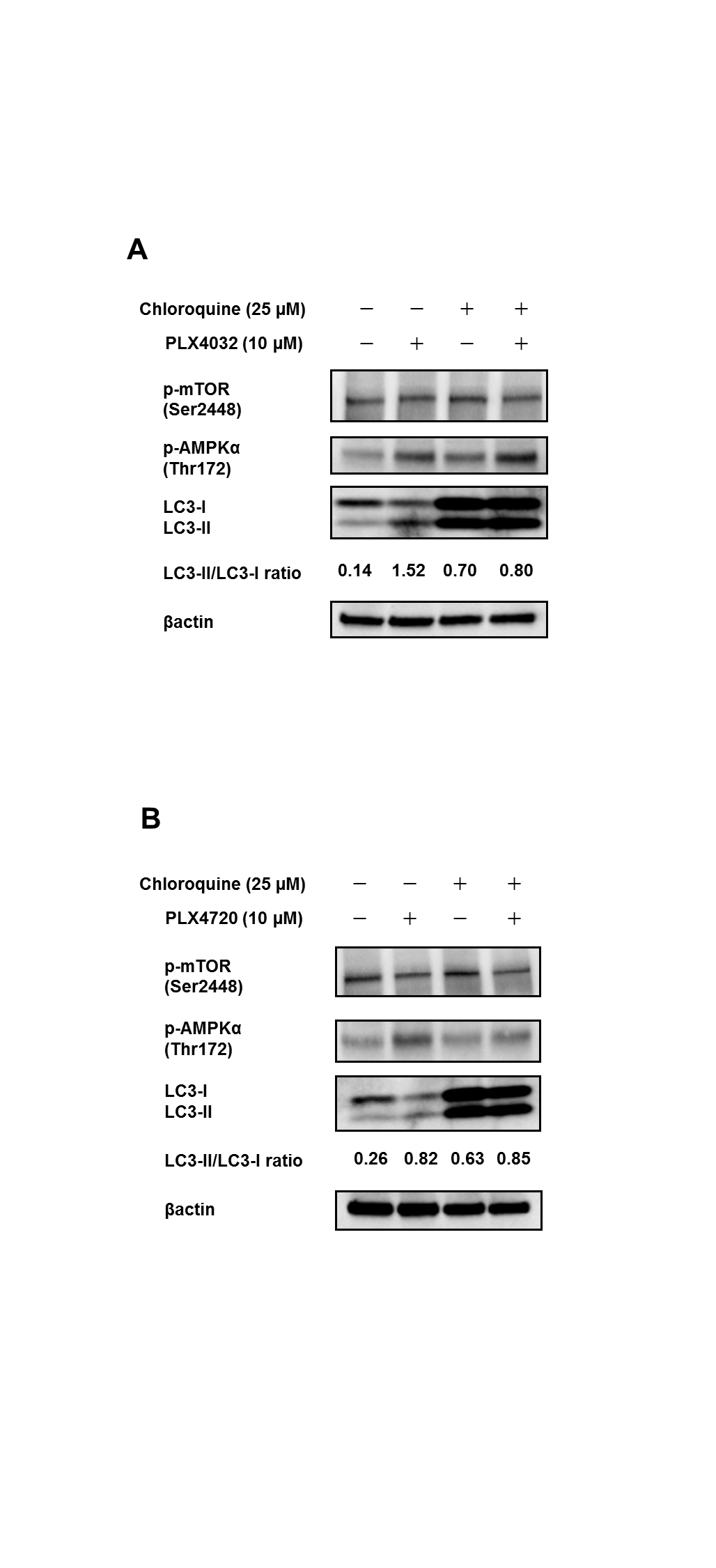
**

**Supplementary Figure 4. Combination treatment-induced autophagy involves in activation of the AMPK pathway, but not activation of the mTOR (Ser2448) pathway.**

(A, B) Western blot assays of phospho-AMPKα (Thr172), phospho-mTOR (Ser2448) and LC3 in HT29 cells after combination treatment with PLX4032 or PLX4720 at 10 µM and CQ at 25 µM for 24 h.

**
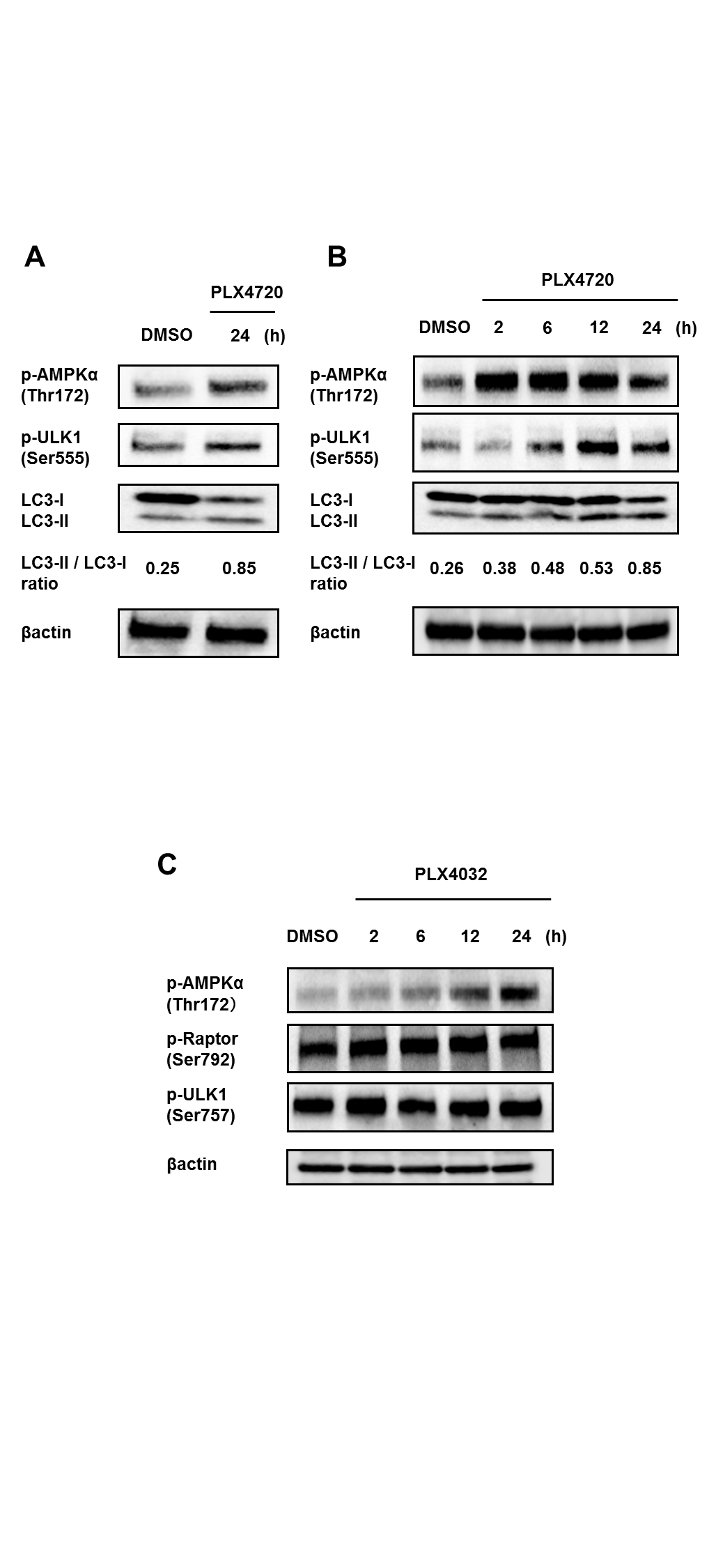
**

**Supplementary Figure 5. Selective BRAF inhibitor-induced autophagy involves with direct AMPK-ULK1 interaction in HT29 cells.**

(A) Western blot assays of phospho-AMPKα (Thr172) and phospho-ULK1 (Ser555) after treatment with DMSO and PLX4720 at 10 µM for 24 h. (B) Western blot assays of phospho-AMPKα (Thr172) and phospho-ULK1 (Ser555) after treatment with DMSO and PLX4720 at 10 µM for 2, 6, 12, and 24 h. (C) Western blot assays of phospho-AMPKα (Thr172), phospho-Raptor (Ser792) and phospho-ULK1 (Ser757) after treatment with DMSO and PLX4032 at 10 µM for 2, 6, 12, and 24 h.

**
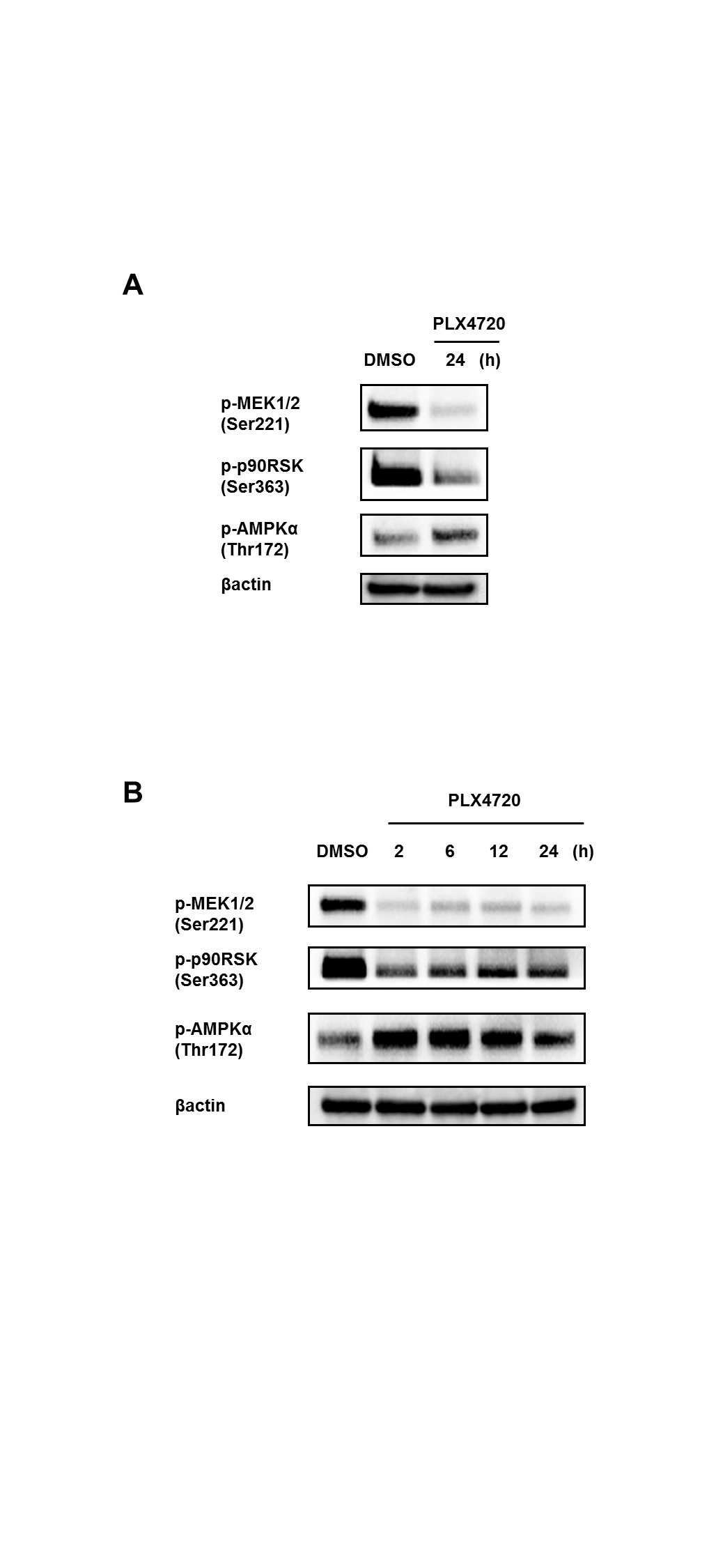
**

**Supplementary Figure 6. Down-regulation of BRAF signaling by PLX4720 also activates AMPK.**

(A) Western blot assays of phospho-MEK1/2 (Ser221), phospho-p90RSK (Ser363) and phospho-AMPKα (Thr172) in HT29 cells after treatment with DMSO and PLX4720 at 10 µM for 24 h. (B) Western blot assays of phospho-MEK1/2 (Ser221), phospho-p90RSK (Ser363) and phospho-AMPKα (Thr172) in HT29 cells after treatment with DMSO for 24 h or PLX4720 at 10 µM for 2, 6, 12, and 24 h.
